# Inter-practitioner comparisons of nerve conduction studies with standardized techniques in normal subjects: Reap as you sow

**DOI:** 10.1097/MD.0000000000019558

**Published:** 2020-04-24

**Authors:** Bing Zhao, Hong-Mei Diao, Qing-Xian Wen, Ke-Xv Sui, Yong-Qing Zhang

**Affiliations:** a Qilu Hospital of Shandong University (Qingdao), Qingdao; b Tengzhou Central People's Hospital, Tengzhou; c Jining No 1 People's Hospital, Jining, China.

**Keywords:** electrodiagnosis, nerve conduction study, normal subject, normal value

## Abstract

This 2-group study was carried out to determine the inter-practitioner difference of nerve conduction studies with standardized techniques.

56 normal subjects of 19 to 49 year-old were recruited, 29, and 27 in the 2 labs respectively. Tests were carried out unilaterally on: 5 motor nerve distal latency, conduction velocities (MNCV) and minimum latency of F wave, 3 sensory nerves with negative amplitude, onset, and peak distal latency, sensory nerve distal latency.

T-test disclosed 4(15.4%) attributes with statistical significance (*P* < .05). They were 2 of 4 (50%) compound motor action potentials, which were ulnar and tibial nerve, and 2 of 6 (33.3%) MNCVs, which were elbow-to-wrist MNCV of median nerve and cross-fibula MNCV of peroneal nerve. No differences were disclosed in motor nerve distal latencys, minimum latency of F waves and all sensory attributes.

Inconsistency pattern of certain attributes were found. This could be explained with the insufficient definition of related techniques.

## 1 Introduction

As an elementary and universal part of electrodiagnostic (EDx) examination, nerve conduction studies (NCs) are commonly used to define the extent and severity of a peripheral neuropathy, identify the specific fiber populations involved, and determine whether the primary pathologic process is axonal or demyelinating. NCs have increasingly been advocated for diagnosis of neuromuscular disorders along with clinical signs and symptoms underlying neurophysiological and neuropathological abnormalities. Most of all, NCs could provide objective, quantitative and reproducible indications of nerve dysfunction. Practically, however, NCs can be challenging with great variability in measurements. Until now, a well-established reference value for routine NCs is still not available.^[1,2]^ And inter-practitioner inconsistency is still the prominent obstacle of clinical practice and therapeutic trials.^[3–5]^ Falck et al proposed that reference values of NCs attributes could be acceptable and understandable across different laboratories when technical factors were “carefully standardized”.^[6]^ Litchy et al verified that reduction of significant inter-practitioner disagreement was achieved when using written instructions and pretrial agreement on techniques in Clinical vs Neurophysiology TRIAL 4 than in Clinical vs Neurophysiology 3, and they confirmed that the variation of inter-practitioner was relate to differences in test performance.^[1]^ Although adopted by some previous studies, the details of “standardized NCs techniques” had not been published. Systematic work has been done by the Normative Data Task Force (NDTF) of the American Association of Neuromuscular and EDx Medicine (AANEM). Techniques that reflect high quality in NCs have been identified from previous studies with uniformed criteria.^[2]^

We follow the hypothesis that variation of inter-practitioner of NCs would be eliminated if the testing is sufficiently well standardized.^[1]^ This study detected the inter-practitioner difference of attributes when NDTF proposed standardized techniques were applied in two different centers with same instrument, which was our prior feasibility examination of yielding a multi-center NCs reference value based on standardized techniques.

## 2 Methods

### 2.1 Subjects recruitment

After obtaining Institutional Review Board approval, subjects were separately recruited to 2 laboratories through advertisements placed on bulletin boards. Exclusion criteria were: age less than 18 or more than 50 years, toxic/metabolic disease, compression neuropathy, symptoms of numbness, tingling, or abnormal sensations, neuromuscular disease, peripheral nerve injury, hereditary neuropathy, radiculopathy, back or neck surgery, cardiac or pulmonary disease, amputation. Each subject read and signed an informed consent.

### 2.2 Demographic and anthropometric factors

For all subjects, age and sex were recorded, height and weight were measured. Body mass index (BMI) was calculated as weight divided by height squared (kg/m^2^).

### 2.3 Subjects preparation

Skin surface temperatures were measured over the dorsum of the hand and foot. If the skin temperatures fell below 32°C for the hand or 31° for the foot, limbs would be warmed as previously described.^[7]^

### 2.4 Nerves, categories and attributes of NCs

Routine motor and sensory studies were performed unilaterally on the following 7 nerves: median, ulnar, peroneal, and tibial motor nerves; median, ulnar and sural sensory nerves. NCs studies were performed in the motor nerves orthodromically and in sensory nerves antidromically. 4 categories of NCs tests were carried out on motor nerves: distal amplitude [compound muscle action potential (CMAP)], motor nerve distal latency (MNDL), motor nerve conduction velocity (MNCV) and minimum latency of F wave (MLFW). And 2 categories were tested on sensory nerves: negative amplitude (sensory nerve action amplitude [negative SNAP]), onset and peak distal latency (sensory nerve distal latency [SNDL]). In all, 26 attributes of each subject were tested in this study: 4 CMAPs; 4 MNDLs; 6 MNCVs and 3 MLFWs; 3 SNAPs, and 6 SNDLs.

### 2.5 Techniques standardization and practitioner training

In pre-trial test, the NDTF edition techniques were revised according with anthropometric characters of normal subjects in our region. In final edition, electrode and stimulator placement, distance measurement was documented with photos of model demonstration. Details of electrode, stimulator placement, distance of recording-electrode to stimulator, display sensitivity, and sweep were listed in Table 1.

**Table 1:**
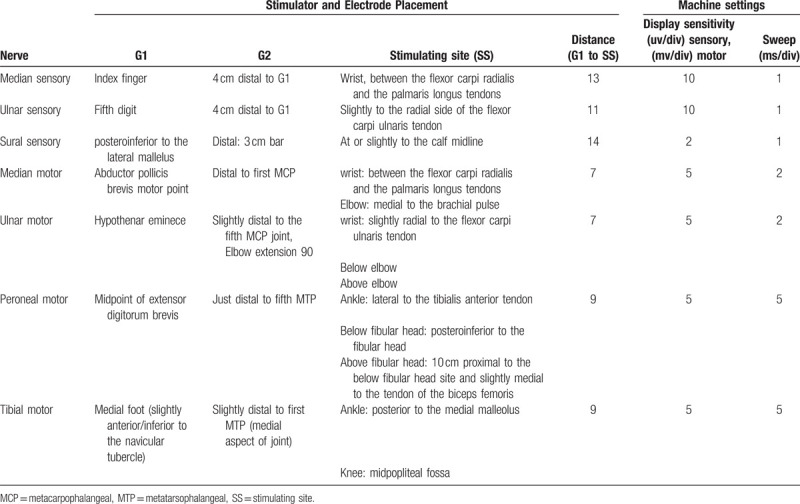
Standardized techniques defined in this study with details in machine settings and stimulator and electrode placement.

Stimulation frequencies were set at 1 Hz for sensory and motor nerve. In order to obtain supramaximal stimulate, the current was increased another 20% when the CMAP or SNAP no longer increased in size with carefully avoiding the co-stimulation of adjacent nerve.

Average techniques with at least 3 measurements were applied in sensory test but not motor studies.

### 2.6 All tests were performed on the Keypoint instrument (Dantec)

Two authors (BZhao, KXSui) served the role of techniques formulating, model demonstrating and practitioner training. The other two authors (HMDiao, QXWen) were trained with the training syllabus, a paper document and photo demonstration of the standardized techniques. One author (YQZhang) served as the modulator of the study to confirm the test trail and data inspection.

Statistical analysis. *T*-test was run to examine the difference of age, BMI difference, and Chi-square test to examine the difference of gender in all branches. *T*-test analysis was performed on all the attributes between groups. The *P* value was set to .05.

## 3 Results

29 and 27 subjects completed all presupposed tests in each laboratory respectively, their demographic and anthropometric characteristics as well as the between-group comparison of age, gender and BMI were listed in Table 2. No significance was disclosed in all these items.

**Table 2:**
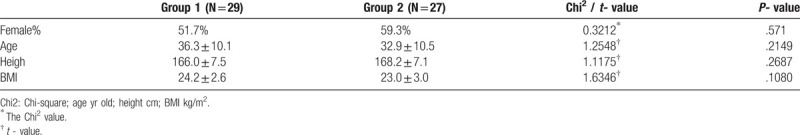
Demographic and anthropometric characteristics of subjects between group.

T-test was performed to examine the difference of all the 26 attributes between groups. 4(15.4%) attributes of motor nerves were revealed with statistical significance (*P* < .05), which were 2 of 4 (50%) CMAPs of ulnar and tibial nerve, and 2 of 6 (33.3%) MNCVs including elbow-to-wrist MNCV of median nerve and cross-fibula MNCV of peroneal nerve. No significance was disclosed in MNDL, MLFW of motor nerve and all attributes of sensory nerve.

## 4 Disscussion

NCs are widely used in the clinical diagnosis, epidemiological surveys,^[8,9]^ and therapeutic trials of neuromuscular disorders.^[10,11]^ Inter-practitioner inconsistencies have been the main obstacle to yielding of a multi-center reference values and longitudinal comparisons in clinical trials, which were believed rooted in the difficulty of documenting and performing standard techniques.

In this 2-group study, standardized techniques were modified from the NDTF of AANEM. Statistical significance was found in 4 (15.4%) motor nerve attributes: 2 (50%) CMAPs of ulnar and tibial nerve and 2 (33.3%) MNCVs which were elbow-to-wrist MNCV of median nerve and cross-fibula MNCV of peroneal nerve. No significance was disclosed in other attibutes.

The first question is whether current standardized techniques brought about more consistencies on the attributes. The data for comparison came from studies with multi-group design on the same subjects. Chaudhry et al tested the consistencies on normal subjects. Each one of the 7 experienced practitioners assessed NCs of the other 4 members. Inconsistency was disclosed in 4 of 12 (33.3%) attributes including 1/2 CMAPs, 1/2 MNCVs, 1/2 of SNAPs and 1/2 MNDLs.^[3]^ The same group then recruited 6 patients with diabetic neuropathy. 6 experienced practitioners performed duplicate NCs on these patients and found inconsistency was same as normal subjects (4/12 attributes), observing in 2/2 CMAPs, 1/2 of SNAPs and 1/2 MNDLs.^[4]^ In CI Phys 3 study, Dyck et al revealed significant interobserver differences in 8 of 8 (100%) attributes on day 1 and 7 of 8 (87.5%) attributes on day 2 without adoping standardized techniques.^[5]^ Specifically, 2/2 CMAP, 2/2 MNDL and 2/2 MNCV, 1/1 SNAP, and 1/1 SNDL were evaluated significantly different on day 1 and only fibular MNCV (1/2 MNCV) didn’t reach significance on day 2.^[5]^ In CI Phys 4 study, 5 of 8 (62.5%) attributes showed statistical significance on day1 and 2 respectively with standardized techniques they defined.^[1]^ They were 2/2 CMAP, 2/2 MNDL and 0/2 MNCV, 1/1 SNAP, and 0/1 SNDL on day 1; 2/2 CMAP, 1/2 MNDL and 1/2 MNCV, 1/1 SNAP, and 0/1 SNDL on day 2. Taking notice of the pattern of attributes with inconsistency in our study, CMAP and MNCV were the only attributes with significant differences. Furthermore, the aboved refereced studies usually evaluated about 8–12 attributes with only ∼2 attributes in each category. Although the attributes were expanded to 26 with 4–6 attributes in each category, relatively high inter-practitioner consistency was definitely maintained in this study.

With the above analysis, the preliminary impression was that standardized techniques could have brought about fewer inconsistencies. However, this conclusion should be carefully declared because our study design inevitably brought less inconsistencies compared to the multi-group design. Even though, we considered the inconsistency pattern of attributes makes more sense than the decreased inconsistency rates. More specifically, this study indicated that standardized techniques could bring about consistency in the MNDL, MLFW, SNAP and SNDL but not CMAP and MNCV. As previous studies concluded that inconsistencies were ascribed to techniques, we hypothesized that a certain aspect of NCs techniques might contribute to a category of attributes, such as, distances of G1-SS to the latencies, distances of proximal-distal SS to the MNCVs, recording-electrode positions and stimulation intensities to the CMAPs and SNAPs.

### 4.1 Reason for the consistency in MNDL, MLFW, SNAP and SNDL

When inspecting the details of standardized technique of this study, notice should be taken of the explicit definition of distance from stimulation-site to record-electrode (see in Table 1, showed as G1-SS). The NDTF adopted the fixed distance in addition to the anatomic landmarks. This would give the consistency of MNDLs, MLFWs and SNDLs, which mostly affected by the distance of G1-SS. Another remarkable pattern was the full-blown consistency in sensory tests where technical factors played a crutical role in the accurate measurement of NCs parameters. It should be emphasized that the potientals of sensory nerves were so delicate to be 3 orders of magnitude smaller than those of motor nerves.^[12–14]^ Thus, standardized techniques provided significant improvement in the proficiency of sensory studies. The case of SNAP would be analyzed when compared with CMAP in the ensuing paragraphs.

**Table 3:**
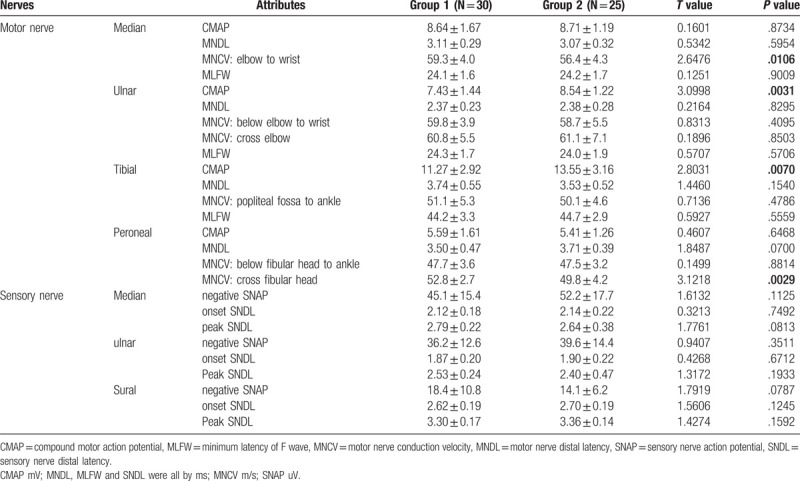
*P* values with robust regression of attributes in each branch.

### 4.2 Why differences exist in CMAPs and MNCVs with current techniques?

When performing the motor NC studies, proper recording electrode placement and stimulation delivery were essential for the measurement of CMAPs. The recording electrode should be placed accurately over the motor endplate of the muscle, which was difficult in one attempt with sometimes further adjustments. In practice, especially when the amplitude is obviously larger than reference value in the mind of an adept practitioner, electrode adjustments were seldomly made. In addition, if the amplitude was large enough, supramaximal stimulation was usually unlikely given especially when the subjects were volunteers. Furthermore, CMAPs were not taken by average method. All discussed above were not the case with SNAPs. Electrode placements were not so technically difficult and critical in antidromic study. The intensities of stimulations were usually less than were required for motor NCs. An average method may also decrease the chance of variation.

The 2 MNCVs with statistical differences were cross elbow CV of ulnar nerve and cross fibula of peroneal nerve. One could easily ascribe the differences of these 2 attributes to the measurements of distance because they appeared to be the most difficult part in practice particularly when standardized techniques could not form explicit regularities in this study. Moreover, the consistency in MNDLs and MLFWs, another 2 EDx markers of myelin function of peripheral nerve, further ruled out the intrinsic variation of subjects themselves and added evidence to the inconsistency of MNCVs.

The origin of NCs variation of inter-practitioner was presumably related to the techniques in a previous study.^[1]^ However, this hypothesis hasn’t been verified. The present study disclosed an inconsistency pattern of attributes with our standardized techniques, which could be interpreted as an insufficient effort to highly standardize certain details of technique.

Standardized techniques bring about consistency of attributes of sensory nerves, MNDLs and MLFWs of motor nerves, but not CMAPs and MNCVs in this study. This pattern of inconsistency could be explained by the insufficient definition of certain techniques. In practice, almost every practitioner has his or her own habits and standards. Among these individual factors, some were easily standardized, such as the distance of G1 to SS and cursor placement while most of the others were more ambiguous or hard to define, such as G1 placement, supramaximal stimulation delivery and distance of the proximal-distal SS of certain nerves. To explicitly define and practically operate every detail was still challenging. Therefore, the inconsistency of CMAPs and MNCVs in this study might ascribe to the lack of detailed descriptions in these specific aspects. However, it is highly promising that inter-practitioner variation could be reduced or diminished and if NCs techniques were sufficiently standardized. In that case, multi-center reference value could be valid just like you reap as you sow.

## Author contributions

B.Z. and Y.Q.Z. conceived and planned the experiments. H.M.D., Q.X.W. and K.X.S carried out the experiments. B.Z., H.M.D., Q.X.W. and K.X.S. contributed to the interpretation of the results. Y.Q.Z. took the lead in writing the manuscript. All authors provided critical feedback and helped shape the research, analysis and manuscript.

Yong-Qing Zhang orcid: 0000-0002-9517-2643.
